# Effects of Combing Group Executive Functioning and Online Parent Training on School-Aged Children With ADHD: A Randomized Controlled Trial

**DOI:** 10.3389/fped.2021.813305

**Published:** 2022-02-11

**Authors:** Liting Chu, Peiying Zhu, Chenhuan Ma, Lizhu Pan, Li Shen, Danmai Wu, Yu Wang, Guangjun Yu

**Affiliations:** ^1^Department of Child Health Care, Shanghai Children's Hospital, Shanghai Jiao Tong University, Shanghai, China; ^2^School of Public Health, Shanghai Jiao Tong University, Shanghai, China; ^3^Clinical Research Center, Shanghai Jiao Tong University Affiliated Sixth People's Hospital, Shanghai, China

**Keywords:** ADHD, non-pharmacological treatment, executive function, online intervention, parent training

## Abstract

**Objective:**

The acceptance of drug treatment for younger children with attention-deficit/hyperactivity disorder (ADHD) in China remains low. Here, we explored the clinical benefits of a non-pharmaceutical intervention method combining a group and executive function training and an online parent training program, termed group executive functioning and online parent training (GEF-OPT), for school-aged students with ADHD through a randomized controlled trial.

**Method:**

A total of 145 children (aged 6–8 years) were formally registered and randomized to the intervention group (*n* = 73) and waitlist group (*n* = 72). The enrolled children received eight sessions of GEF-OPT treatment, which consists of a hospital-based children executive function (EF) training program and an online parent training program. Treatment outcome was assessed by a parent/teacher report questionnaire and neurophysiological experiment.

**Results:**

After eight sessions of intervention, children in the intervention group showed a significant improvement in inattentive symptom compared to the waitlist group (14.70 ± 4.35 vs. 16.03 ± 2.93; *p* = 0.024), but an insignificant difference in hyperactive-impulsivity (9.85 ± 5.30 vs. 10.69 ± 5.10; *p* = 0.913). Comorbid oppositional defiant disorder was significantly reduced in the intervention group (7.03 ± 4.39 vs. 8.53 ± 4.41; *p* = 0.035). Children in the intervention group had greater reduction in the scores of behavioral regulation index (inhibition, emotional control) and metacognition index (working memory, planning/organization, monitoring) in executive function than those in the waitlist group (*p* < 0.05). Significant effects were also found in learning problem of Weiss Functional Impairment Scale–Parent form and parental distress between two groups at post-treatment (*p* < 0.05). In line with this, the result of go/no-go task showed significant improvements in accuracy change (4.45 ± 5.50% vs. 1.76 ± 3.35%; *p* = 0.001) and reaction time change (47.45 ± 62.25 s vs. 16.19 ± 72.22 s; *p* = 0.007) in the intervention group compared with the waitlist group.

**Conclusion:**

We conclude that participants in the GEF-OPT program improved outcomes for inattentive symptom, executive function, learning problems, and parental distress. GEF-OPT is a promising non-pharmaceutical therapeutic option for younger children.

**Trial Registration:**

ChiCTR2100052803.

## Introduction

Attention-deficit/hyperactivity disorder (ADHD) is a common neurodevelopmental disorder in childhood, characterized by hyperactivity, impulsivity, and inattention that are not commensurate with the developmental level. ADHD not only impedes the development of children's learning and social abilities but also brings a heavy burden on their families and society (1). A meta-analysis indicates that the prevalence of ADHD among children and adolescents in China is 6.26%, generally consistent with the worldwide prevalence (2). Medical treatment (methylphenidate, atomoxetine, etc.) can relieve the core symptoms of ADHD (3–6); however, a considerable proportion of patients fail to tolerate or respond to the stimulant treatment (7). Further, the evidence that drug therapy can prevent a series of comorbidities in later childhood or adulthood is lacking (5, 8). Recently, many treatment guidelines emphasize the importance of multimodal treatment for ADHD, which consists of combining drug treatment and non-drug treatment (i.e., parent training and social skills training) (9–11).

Executive function (EF) deficits are major contributors to poorer outcomes in ADHD patients (12, 13), which have been directly related to impairments in academic, interpersonal, and social functioning (14, 15). EF is the high-level cognitive function of the central nervous system that promotes new behaviors (16). ADHD patients with deficiencies in EF show functional impairments, including inhibition, planning, work memory, plan organization, and cognitive flexibility (17). These impairments associated with ADHD highlight the importance of the early and appropriate interventions in improving the developmental trajectories (18). Group-based EF training is currently recommended to help children with ADHD symptoms. Lan et al. (19) compared the effects of group EF training with social skills training in children with ADHD and found that EF training produced more effective and lasting changes on peer relationship difficulties. Qian et al. (20) found 33 school-aged students who benefitted from ecological executive skills training, and these children exhibited less core symptoms 1 year later, compared with the control group. Therefore, it is necessary to give EF training for school-aged children with ADHD.

Parent management training (PMT) is a psychosocial intervention program that allows the parents of ADHD children to apply the behavior management methods to effectively manage children's challenging behaviors (21). These methods are favored by parents who are resistant to medication (22). These parent training programs include Incredible Years (23), the New Forest Parenting Program (24), and Positive Parenting Program (25), some of which have achieved positive therapeutic effects (26). Most efficacious studies are traditional on-site interaction (23, 27), which refers to parents receiving training lessons from doctors or therapists face to face, then conducting behavioral training for children at home. However, this type of training is often hindered by time and traffic restraints. Retention in Barkley's study is poor, with only 25% of parents attending more than 4 of 14 sessions (28). Moreover, the benefit of parent training intervention in long-term follow-ups has generally not been demonstrated. In a notable exception, Shelton's research proposed that the effects of parent management training did not persist at a 2-year follow-up (29). Coincidentally, some studies also pointed out that parent training and pharmacological treatment are not so effective for children with ADHD and that parental compliance is very important (30, 31). Currently, the rapid development of digital health has made it possible for the Internet-based parental training. Studies have confirmed that digital health intervention provides patients with high accessibility, scalability, and cost-effectiveness while still improving patient outcomes (32). For example, Franke and colleagues demonstrated that an online parenting program is an effective intervention for preschool children (33). The efficacy of a web-assisted self-help parenting program was also verified by a large sample size (34). Thus, it can be considered that web-based parenting training is a feasible measure in ADHD intervention.

Given that parents are more willing to accept non-pharmacological interventions for school-aged children with ADHD, we explored the clinical benefits of non-pharmacological interventions combining the group executive functioning and online parent training (GEF-OPT) for ADHD children aged 6–8 years old. To do so, two hypotheses were examined. The first is whether the intervention group (parents and teachers) reports lower levels of child core ADHD symptoms after intervention compared with parents/teachers in the waitlist group. The second is whether the non-pharmacological interventions show some key improvements over the waitlist group, including: (a) improvements in executive functioning; (b) improvements in peer relationship, learning, and social function; and (c) lower levels of parental pressure and anxiety. Our research was performed within a hospital-based group training center plus online platforms in order to facilitate child intervention and parent training. A randomized controlled trial (RCT) was conducted to investigate the training effects of GEF-OPT after intervention.

## Methods

### Study Design and Population

All participants were from primary schools in Putuo District, Shanghai, China. Similar to our previous study (35), an invitation and information letter was sent to in-house healthcare professionals and the headmasters of involved schools, informing them about the study. The hospital pediatricians then conducted an online meeting about the purpose of this project and the type of intervention for both teachers and parents. Parents who wanted to participate in this project would contact the research assistant, and then be registered in a WeChat group, where all related matters and screening system would be informed.

**Figure 1:**
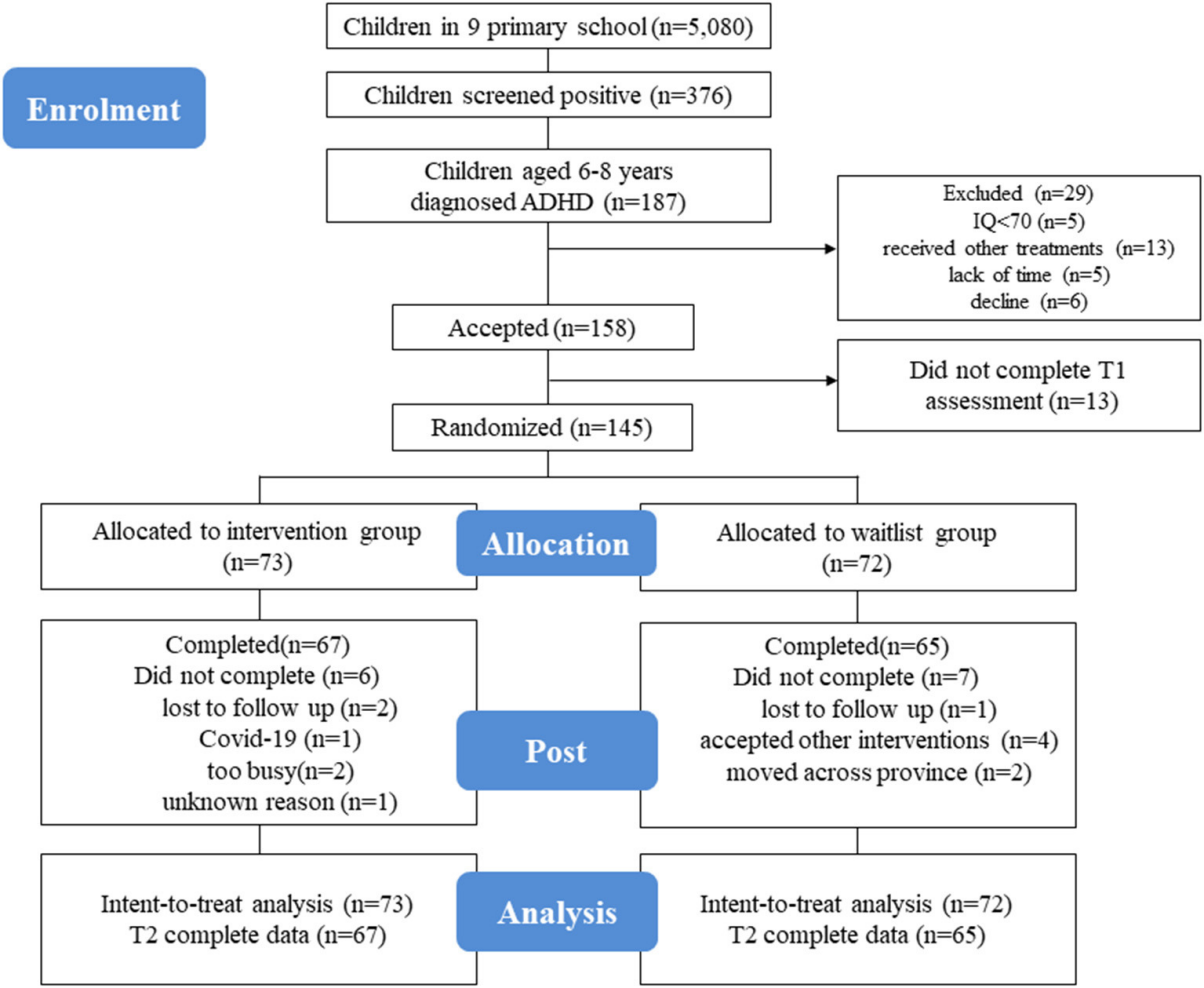
This figure shows the consort diagram of the RCT. All outcomes were measured before and after the intervention for both groups. The waitlist group received the same intervention after the second assessment. Six participants in the intervention group (two lost to follow-up, one was unwilling to go to the hospital due to COVID-19, two were too busy, and one of an unknown reason) and seven participants in the waitlist group (one lost to follow-up, four accepted other interventions, and two moved across a province) dropped out of the study.

Participants, recruited between January 2021 and June 2021, are screened for ADHD *via* a mobile app Swanson Nolan and Pelham, Version IV (SNAP-IV) Scale. Parents would directly receive the positive or negative results after completing the electronic scale, and then, they could decide on their own whether to take their children to Shanghai Children's Hospital for diagnosis. After 1 month of parents self-filling the electronic scale, pediatricians identified 187 6–8-year-old children diagnosed with ADHD according to the DSM-5 criteria by detailed medical history collection and behavioral observation (36). Research assistants sent the project invitation and informed consent form to the parents of these children. Eventually, there were 145 ADHD children who participated in this study. Scale evaluation was done by those who were familiar with children's daily life at home and school, mainly parents and class teachers. Assessment took place at two time points: at pre-intervention (T1) and post-intervention (T2; 8 weeks after T1). After T1 assessment, families were randomly allocated to the intervention or waitlist group. The waitlist group received the intervention after T2 assessment. The consort diagram of each stage of RCT and drop-out reasons is shown in Figure 1.

### Inclusion and Exclusion Criteria

Children were newly diagnosed with ADHD, following the criteria of *Diagnostic and Statistical Manual of Mental Disorders, fifth edition* (DSM-5) (36), ranging from 6 to 8 years old. IQ should be 70 or above established with the Wechsler Intelligence Scale for children–fifth edition (WISC-V) (37). Moreover, parents or primary caregivers did not want to receive drug therapy, could read and write the Chinese language, were legally able to sign informed consent, and signed the informed consent.

Children with autism spectrum disorder, schizophrenia, epilepsy, head injury, or verified neurological disorder, intellectual disability (IQ <70, based on WISC-V) (38), and sensory impairment (hearing/vision problems) and those receiving other ADHD treatments were excluded. Neither the intervention nor waitlist group were treated with medication.

### Randomization and Blinding

The participants who met all eligibility criteria and provided written informed consent were randomly assigned (1:1) to receive intervention or wait to do intervention using a computer-generated randomization sequence. Randomization was done by research staff using statistics software (SAS 9.4, SAS Institute, Cary, NC, USA). Given the nature of this study, participants could not be blind to their assigned group, so the participants and pediatrician were aware of group allocation. Other research staff were blind to the group. Analyses were done by a statistician masked to group allocation.

### Online Parent Training and Group Executive Function Training

We provided a multimodal treatment for children and parents in the intervention group. The GEF-OPT in this study was based on Training Executive, Attention, and Motor Skills (TEAMS) (39), which was modified to be more suitable for Chinese elementary school families.

The training program consisted of eight 90-min sessions, composed of separate child and parent groups (four-to-six families per group). Before treatment, parents were told to help children prepare a notebook, pencil, and eraser. The children took part in group EF training in a clinical setting, and parents received OPT *via* Voov Meeting (computer, tablet, or mobile phone). Parents had a 30-min lesson to learn about ADHD and behavioral management skills and conduct behavioral management while assisting children in completing homework after EF training class. Child groups were led by a team of three staff: typically one senior psychologist and two graduate students. Parent groups were run by three professional pediatricians specializing in child healthcare. The outline for each session is presented in Table 1.

**Table 1:** Contents of GEF-OPT.

**Week**	**Targeted executive function**	**Part of group executive function training**	**Online parent training**
1	Sustained attention	Commitment: Each child was asked to tell a class rule and then wrote it down or express with pictures in the notebook. Visual tracking: The therapist took out three playing cards and put them face up in a row, and asked children to choose one (for example, spades A). Then the therapist put them back to its original position, asked children to focus on the card, and moved the card quickly from side to side. After several moves, children were asked to point out the position of spades A from the three playing cards. The number and type of cards would be changed.	Knowledge about ADHD and methods of family attention training
2	Planning and time management	Schedule: The therapist taught children planning and time management skills and gave each child a timetable as well as asked them to formulate the time they spend on necessary events and other activities for the following week (homework, tutoring class, extracurricular activities, etc.). Children were required to complete the weekly schedule.	Help children manage time and supervise them to complete each task according to the schedule
3	Organization skills	Room and desk organization: Children should be first asked to distinguish clean and cluttered room and desk. The therapist used teaching aids to classify and organize possessions in the room and study with children. Homework was to tidy up the room and desk, and complete a task list for hosting a birthday party.	Learn to mobilize children's enthusiasm and praise them in time
4	Inhibition	Simon says: One child acted as Simon and gave instructions to other children (nodding, stomping, touching nose, etc.). When he started with “Simon says,” the rest of children needed to follow instructions, otherwise they should keep still.	Learn behavioral strategies such as positive reinforcement and punishment to manage conduct problems
5	Working memory	Sherlock: The therapist gave out 8 cards with arrows of different clues (daily necessities, fruits, animals, clothing, etc.). Children needed to remember the evidence on the cards. Then the therapist turned the card face down and picked up the doll. The doll moved according to the arrow and the number of steps on the card. If the child answered correctly and the card the doll stayed on was turned over, the child would get this card.	Strategies for effective learning skills and communication with teachers
6	Spatial intelligence	Matchmaker: The therapist gave a card surrounded by 10 blocks (from easy to difficult). Children needed to flip 5 long blocks in the shortest time to match the corresponding pattern.	Guidelines for giving effective instructions
7	Cognitive flexibility	My first journey: The therapist taught children to understand the map of China. Four city tickets were randomly selected on the table. Each child had another four city tickets, then took turns rolling the dice, and chose the route according to the color of the dice and city tickets. When the arrival city was the same as the four tickets on the table, the child could get the ticket of the stated characteristics of the city.	Games of improving parent-child relationship and methods for stress management
8	Consolidate and summarize	Consolidate and reinforce the poorly-performed projects completed before. Children shared their positive changes and received rewards.	Questions and answers Review and identified obstacles resolution

### Sample Size Calculation

The primary endpoint was the total scores of parents reported SNAP-IV scale after intervention. It was estimated that a total sample of 140 (1:1) would be sufficient to demonstrate a statistically significant difference between the intervention and waitlist group with 90% power and an alpha of 0.05 and expected dropout of 10%.

### Statistical Analysis

Data analyses were performed with SAS v9.4 (SAS Institute). The difference between two groups was assessed by an independent sample *t*-test for continuous data and chi-square test for categorical data. Analysis of covariance (ANCOVA) was used to compare the intervention effects between two groups with pre-intervention data as covariates. The magnitude of effect sizes was expressed in Cohen's *d*, which is computed by comparing the change scores between intervention and waitlist groups and dividing them by the pooled standard deviation (SD) of change scores. Data were shown as mean ± SD and frequency (percentage). Missing data were imputed by last observation carried forward (LOCF) and followed by intention-to-treat (ITT) analysis guidelines. All statistical analyses were two-tailed, and *P* < 0.05 was considered statistically significant.

### Questionnaires and Experiments

#### Swanson Nolan and Pelham, Version IV Rating Scale

The SNAP- IV is composed of 26 items using a four-point scale ranging from 0 to 3, including three subscales: inattention, hyperactivity, and Oppositional-defiant disorder (ODD). A higher score indicates greater levels of symptoms. This scale was reported to have good reliability and validity (40). The SNAP- IV was completed by parents and teachers via a mobile app, with ~15 min to complete. The primary outcome in this study was the total scores of the parent-rated SNAP-IV scale between the intervention and waitlist group at T2.

#### Behavior Rating Inventory of Executive Function-Parent Form (BRIEF)

The Behavior Rating Inventory of Executive Function-Parent Form (BRIEF) is a questionnaire for parents of school-aged children that enables professionals to assess EF behaviors at home. It contains 86 items within eight theoretically and empirically derived clinical scales that measure the different aspects of EF: Inhibition, Shift, Emotional Control, Initiate, Working Memory, Planning/Organization, Organization of Materials, and Monitor (41). The Chinese version of this scale has good reliability and validity and is suitable for those with a Chinese cultural background (42).

#### Go/No-Go Task

Go/No-Go task is frequently used to investigate response inhibition (43). In this study, the test was performed according to Monden's research (44), which includes six block sets, namely, alternating Go, No-Go, and Go/No-Go blocks. In the Go block, a child was asked to recognize a picture of elephants and tigers (100%) and then quickly pressed the space bar. In the Go/No-Go block, a child was provided with lion pictures (50%) that require a button press and giraffe pictures that do not require a button press (50%). Each block lasted for 24 s. Before each block, there were 3 s of instruction in Chinese telling children to press the space bar when they saw elephants and tigers, pressed the space bar when they saw lions, and not press any button when they saw giraffes. The total block setting time was 54 s, and the overall session time was about 6 min.

The accuracy (RC) and reaction time (RT) of each child were recorded for the behavior analysis. The Go/No-Go task of this experiment were presented on a 24-in. computer screen by E-Prime 2.0 software. The distance between child's eyes and the computer screen is approximately 50 cm. Before collecting data, all participants must receive guidance and actually perform several experimental tasks, and the examiner observed the completion of participants to ensure that the participants correctly understand the experimental tasks.

#### Weiss Functional Impairment Scale–Parent Form

The Weiss Functional Impairment Scale–Parent form (WFIRS-P) is a social function assessment tool compiled based on the characteristics of ADHD. It is used by parents based on children's emotional and behavioral aspects in the recent month. The scale has a total of 50 items, including six subscales of family, learning and school, life skills, children's self-concept, social activities, and risky activities. Previous research showed that the WFIRS-P of Chinese version has good reliability and validity, with an internal consistency of 0.70–0.92, and a test–retest reliability of 0.61–0.87 (42).

#### Parenting Stress Index

Parenting Stress Index (PSI) refers to the difficulties, anxiety, tension, and other pressures that parents have in the process of fulfilling their parental roles and parent–child interactions. There are 36 items in total, including three subscales: parenting distress, dysfunctional interaction, and child difficulty. High scores show great levels of parenting stress. The PSI has shown adequate reliability and high validity in Chinese children (45).

## Results

### Descriptive Analyses

A total of 187 (3.7%) students aged 6–8 years were diagnosed with ADHD. After exclusion, 145 children were enrolled and randomized to the intervention group (*n* = 73) and waitlist group (*n* = 72). Attrition included six children in the intervention group (two were lost to follow-up, one withdrew due to COVID-19, two were too busy, and one with an unknown reason) and seven waitlist group children (one was lost to follow-up, four accepted other interventions, and the other two moved out of a province) (Figure 1). Eventually, there were 132 families (91.0%) that completed the study at T2 (Figure 1).

Analyzing the basic demographic information, including the age, IQ, gender, ADHD subtypes, comorbidities, and family status, of the intervention and waitlist groups, did not reveal a significant difference between these two treatment conditions on any of the demographics or baseline variables (Table 2, *P* > 0.05).

**Table 2:** Demographic characteristics of the intervention group and the waitlist group.

**Variable**	**Intervention** **(*n* = 73)**	**Waitlist** **(*n* = 72)**	***t/χ^2^***	***P***
**Age (years), mean** **±SD**	7.10 ± 0.47	7.04 ± 0.61	0.666	0.506
**IQ, mean** **±SD**	97.01 ± 17.31	96.36 ± 12.23	0.262	0.794
**Gender**, ***n*** **(%)**			0.667	0.414
Boy	57 (78.1)	52 (72.2)		
Girl	16 (21.9)	20 (27.8)		
**ADHD subtype**, ***n*** **(%)**			1.002	0.606
Inattentive	45 (61.6)	42 (58.3)		
HI	8 (11.0)	12 (16.7)		
Combined	20 (27.4)	18 (25.0)		
**Comorbidity**, ***n*** **(%)**				
ODD	15 (20.5)	13 (18.1)	0.145	0.704
Anxiety and depression	2 (2.7)	4 (5.6)	0.725	0.395
**Family structure**, ***n*** **(%)**			1.242	0.265
Core family	40 (54.8)	46 (63.9)		
Non-core family	33 (45.2)	26 (36.1)		
**Family annual income, yuan** ***n*** **(%)**			2.687	0.261
~100,000	9 (12.3)	10 (13.9)		
100,000–200,000	19 (26.0)	27 (37.5)		
200,000~	45 (61.6)	35 (48.6)		
**Parental relationship**, ***n*** **(%)**			0.090	0.764
Harmony	49 (67.1)	50 (69.4)		
General	24 (32.9)	22 (30.6)		
**Father's education**, ***n*** **(%)**			1.602	0.449
College~	12 (16.4)	16 (22.2)		
High school-college	48 (65.8)	40 (55.6)		
~Junior high school	13 (17.8)	16 (22.2)		
**Mother's education**, ***n*** **(%)**			0.510	0.775
College~	9 (12.3)	9 (12.5)		
High school-College	53 (72.6)	49 (68.1)		
~Junior high school	11 (15.1)	14 (19.4)		
**Parent-child communication time**, ***n*** **(%)**			0.222	0.638
<3 d/w	2 (2.7)	3 (4.2)		
≥3 d/w	71 (97.3)	69 (95.8)		
**Parent-child outdoor activities**, ***n*** **(%)**			2.846	0.092
<3 d/w	43 (58.9)	52 (72.2)		
≥3 d/w	30 (41.1)	20 (27.8)		
**Children's exposure to electronic screens time**, ***n*** **(%)**			5.239	0.073
1 h/d~	38 (52.1)	27 (37.5)		
0.5–1 h/d	19 (26.0)	17 (23.6)		
~0.5 h/d	16 (21.9)	28 (38.9)		

*ADHD, Attention deficit hyperactivity disorder; IQ, Intelligence quotient; HI, Hyperactive- impulsivity; ODD, Oppositional-defiant disorder; SD, Standard deviation*.

### Effects of GEF-OPT by SNAP-IV Scales

For assessing the changes in ADHD symptoms, we applied a Chinese version of SNAP-IV, which has good reliability and validity (46). As shown in Table 3, the primary outcome was presented as SNAP-IV scales of the core items. After adjusting the baseline scale data of pre-intervention, the significant difference could be observed in parent-rated inattentive [*F*_(1, 143)_ = 5.17, *P* = 0.024, *d* = 0.27] and ODD [*F*_(1, 143)_ = 4.55, *P* = 0.035, *d* = 0.27] as well as teacher-rated inattentive [*F*_(1, 143)_ = 13.23, *P* < 0.001, *d* = 0.53], ODD [*F*_(1, 143)_ = 13.05, *P* < 0.001, *d* = 0.53], and total score [*F*_(1, 143)_ = 14.76, *P* < 0.001, *d* = 0.43]. Both Hyperactive-impulsivity (HI) and the total score in parent-rated SNAP-IV scales did not show significant treatment effects, while only HI in teacher-rated SNAP-IV scales was not statistically different between two groups.

**Table 3:** Effects of GEF-OPT by SNAP-IV scales.

**Scales**	**Intervention group (n** **=** **73)**		**Waitlist group (n** **=** **72)**		***F***	***P***	***d* [95% CI]**
	**Pre**	**Post**	**Pre**	**Post**			
**SNAP-IV, parent rated**							
Inattentive	15.66 ± 3.99	14.70 ± 4.35	15.86 ± 4.03	16.03 ± 2.93	5.17	0.024	0.27 [−0.06, 0.60]
HI	11.47 ± 5.19	9.85 ± 5.30	12.58 ± 5.52	10.69 ± 5.10	0.01	0.913	−0.41 [−0.69, −0.14]
ODD	8.53 ± 4.78	7.03 ± 4.39	8.93 ± 3.94	8.53 ± 4.41	4.55	0.035	0.27 [−0.03, 0.57]
Total score	35.66 ± 9.79	31.58 ± 11.32	37.38 ± 10.74	35.25 ± 10.44	3.34	0.070	0.06 [−0.21, 0.33]
**SNAP-IV, teacher rated**							
Inattentive	16.19 ± 2.99	14.56 ± 3.96	15.90 ± 4.05	16.06 ± 2.74	13.23	<0.001	0.53 [0.24, 0.82]
HI	12.74 ± 4.10	10.64 ± 4.79	12.46 ± 4.53	11.28 ± 4.16	2.59	0.110	−0.09 [−0.36, 0.18]
ODD	9.60 ± 3.89	7.86 ± 3.93	8.92 ± 3.79	8.90 ± 3.62	13.05	<0.001	0.53 [0.28,0.78]
Total score	38.53 ± 7.76	33.07 ± 10.06	37.28 ± 10.54	36.24 ± 9.48	14.76	<0.001	0.43 [0.17, 0.69]

*All data are shown as mean ± SD*.

*SNAP-IV, Swanson Nolan and Pelham, Version IV Rating Scale; HI, Hyperactive-impulsivity; ODD, oppositional-defiant disorder; SD, Standard deviation*.

### Effects of GEF-OPT by BRIEF Scales

To assess the EF behaviors of patients at home, the BRIEF scales were then analyzed. There were significant effects in inhibition [*F*_(1, 143)_ = 21.85, *P* < 0.001, *d* = 0.69], emotional control [*F*_(1, 143)_ = 7.24, *P* = 0.008, *d* = 0.33], working memory [*F*_(1, 143)_ = 6.81, *P* = 0.010, *d* = 0.27], planning/organization [*F*_(1, 143)_ = 5.10, *P* = 0.025, *d* = 0.32], monitor [*F*_(1, 143)_ = 7.45, *P* = 0.007, *d* = 0.34], behavioral regulation index [*F*_(1, 143)_ = 14.77, *P* < 0.001, *d* = 0.42], metacognition index [*F*_(1, 143)_ = 7.39, *P* = 0.007, *d* = 0.30], and total score [*F*_(1, 143)_ = 12.67, *P* = 0.001, *d* = 0.32]. Although the subscale scores of waitlist group also decreased at T2, the effects of intervention group were improved more significantly than that of the waitlist group (Table 4).

**Table 4:** Effects of GEF-OPT by BRIEF scales.

**Scales**	**Intervention group (*n* = 73)**		**Waitlist group (*n* = 72)**		***F***	***P***	***d* [95% CI]**
	**Pre**	**Post**	**Pre**	**Post**			
**BRIEF**							
Inhibition	19.88 ± 5.44	17.21 ± 4.37	19.85 ± 4.12	19.44 ± 4.58	21.85	<0.001	0.69 [0.43,0.95]
Shift	13.00 ± 2.76	13.07 ± 2.69	13.85 ± 2.53	13.60 ± 2.70	0.00	0.982	0.00 [−0.33,0.33]
Emotional control	17.36 ± 4.80	15.82 ± 4.27	18.36 ± 4.47	17.78 ± 4.59	7.24	0.008	0.33 [0.11,0.55]
Initiate	15.33 ± 2.98	14.79 ± 2.80	15.29 ± 2.84	14.99 ± 3.16	0.34	0.562	0.06 [−0.21,0.33]
Working memory	22.47 ± 3.60	21.22 ± 4.12	23.54 ± 3.46	23.32 ± 3.80	6.81	0.010	0.27 [0.01,0.54]
Planning/organization	25.71 ± 4.67	24.42 ± 4.62	25.63 ± 4.84	25.50 ± 4.50	5.10	0.025	0.32 [0.09,0.56]
Organization of materials	12.08 ± 2.31	11.52 ± 2.51	12.00 ± 2.44	11.93 ± 2.17	2.89	0.091	0.28 [0.03,0.53]
Monitor	19.53 ± 3.14	17.92 ± 3.09	19.64 ± 2.95	19.01 ± 2.86	7.45	0.007	0.34 [0.05,0.63]
BRI	50.23 ± 10.41	46.10 ± 8.68	52.06 ± 9.17	50.82 ± 10.41	14.77	<0.001	0.42 [0.21,0.63]
MI	95.12 ± 13.38	89.88 ± 14.15	96.10 ± 15.03	94.75 ± 14.93	7.39	0.007	0.30 [0.07,0.53]
Total score	145.36 ± 20.71	135.97 ± 19.83	148.15 ± 23.23	145.57 ± 24.33	12.67	0.001	0.32 [0.09,0.54]

*All data are shown as mean ± SD*.

*BRIEF, Behavior Rating Inventory of Executive Function-Parent Form; BRI, Behavioral Regulation Index; MI, Metacognition Index; SD, Standard deviation*.

### Effects of GEF-OPT by WFIRS-P and PSI Scores

To further confirm the beneficial effects of GEF-OPT, we assessed the WFIRS-P and PSI scores. In line with the BRIEF scales, significant differences were also observed in the Learning and School subscale, [*F*_(1, 143)_ = 8.52, *P* = 0.004, *d* = 0.60], and the total score of WFIRS-P [*F*_(1, 143)_ = 6.99, *P* = 0.009, *d* = 0.30] between GEF-OPT and waitlist groups (Table 5). At T2, parents in the GEF-OPT group showed a significantly greater decrease in parenting distress [*F*_(1, 143)_ = 28.45, *P* < 0.001, *d* = 0.73], dysfunctional interaction [*F*_(1, 143)_ = 37.72, *P* < 0.001, *d* = 0.98], child difficulty [*F*_(1, 143)_ = 14.39, *P* < 0.001, *d* = 0.91], and the total score of PSI [*F*_(1, 143)_ = 48.75, *P* < 0.001, *d* = 1.20] than their counterparts in the waitlist group (Table 5).

**Table 5:** Effects of GEF-OPT by WFIRS-P and PSI scores.

**Scales**	**Intervention group (*n* = 73)**		**Waitlist group (*n* = 72)**		***F***	***P***	***d* [95% CI]**
	**Pre**	**Post**	**Pre**	**Post**			
**WFIRS-P**							
Family	8.03 ± 4.14	6.84 ± 3.61	8.50 ± 3.64	7.69 ± 3.81	1.43	0.233	0.11 [−0.25, 0.48]
Learning and school	6.25 ± 3.50	5.23 ± 2.94	5.26 ± 3.12	6.14 ± 3.39	8.52	0.004	0.60 [0.27, 0.94]
Life skills	9.55 ± 3.86	9.18 ± 3.32	9.40 ± 4.03	9.64 ± 4.85	0.82	0.365	0.17 [−0.15, 0.48]
Self-concept	2.26 ± 1.91	2.07 ± 1.78	2.10 ± 1.46	2.07 ± 1.35	0.03	0.855	0.11 [−0.31, 0.53]
Social activities	5.81 ± 3.69	4.85 ± 2.99	5.94 ± 2.87	5.63 ± 3.42	2.05	0.155	0.20 [−0.21, 0.61]
Risky activities	2.91 ± 2.09	2.64 ± 2.25	3.18 ± 1.82	2.97 ± 2.05	0.35	0.553	0.05 [−0.30, 0.39]
Total score	34.80 ± 12.79	30.81 ± 11.47	34.39 ± 12.50	34.14 ± 10.49	6.99	0.009	0.30 [0.03, 0.56]
**PSI**							
Parenting distress	27.78 ± 4.87	25.16 ± 4.17	28.79 ± 4.38	28.51 ± 4.03	28.45	<0.001	0.73 [0.43, 1.03]
Dysfunctional interaction	28.78 ± 5.98	24.99 ± 4.77	28.72 ± 5.93	28.29 ± 4.41	37.72	<0.001	0.98 [0.67, 1.29]
Difficult child	27.74 ± 6.14	25.52 ± 4.96	27.21 ± 5.54	27.08 ± 5.38	14.39	<0.001	0.91 [0.65, 1.16]
Total score	84.30 ± 13.11	75.67 ± 10.23	84.72 ± 13.71	83.89 ± 11.27	48.75	<0.001	1.20 [0.89, 1.50]

*All data are shown as mean ± SD*.

*WFIRS-P, WEISS Functional Impairment Scale-Parent form; PSI, Parent Stress Index; SD, Standard deviation*.

### Effects of GEF-OPT by Go/No-Go Task Analysis

**Figure 2:**
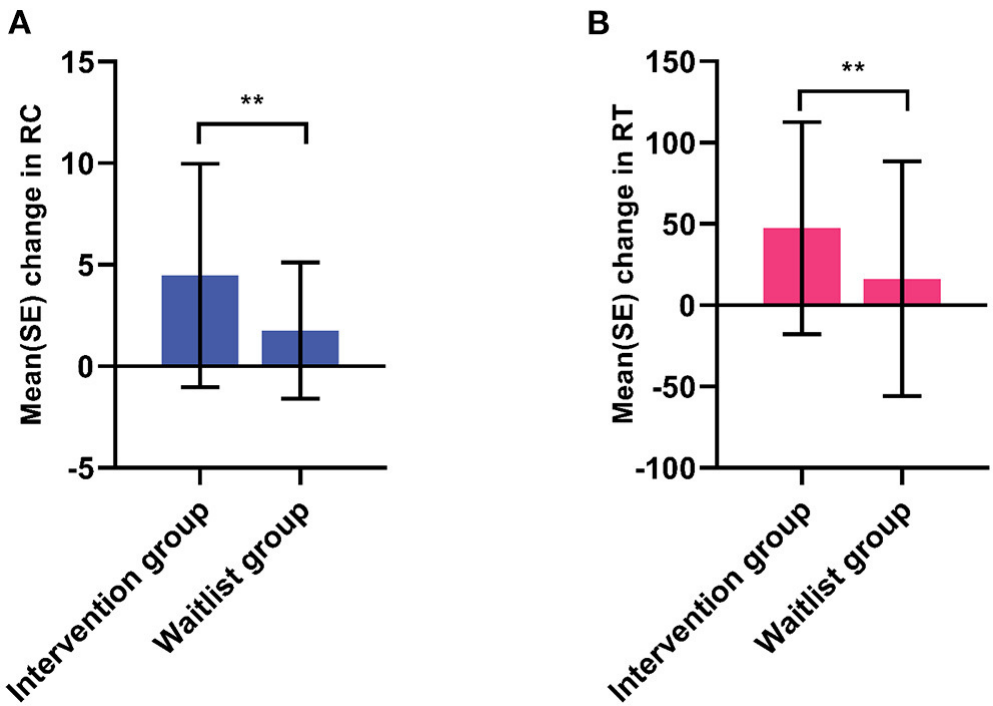
Analysis of Go/No-Go task. The mean change of accuracy **(A)** and reaction time **(B)** of enrolled students in GEF-OPT intervention group (*n* = 73) and waitlist group (*n* = 72) were analyzed using Student *t*-test. Significant differences were found in difference of accuracy (*P* = 0.001) and reaction time mean change (*P* = 0.007) between intervention group and waitlist group. RC, accuracy; RT, reaction time; **P < 0.01.

The Go/No-Go task is frequently used to investigate response inhibition (43). We then set out to assess the effect of GEF-OPT intervention on enrolled children using the Go/No-Go task. There was no significant difference in RC between the intervention group (85.67 ± 6.75) and waitlist group (86.12 ± 8.08) at T1, while a significant difference was found in the increase of RC in two time points, with 4.45 ± 5.50 in the intervention group and 1.76 ± 3.35 in the waitlist group (*t* = 3.561, *P* = 0.001) (Figure 2A). The RT of the intervention group was 478.33 ± 56.46 at T1 and 430.87 ± 54.21 at T2, while the waitlist group was 483.95 ± 43.70 and 467.75 ± 53.90. The reduction of RT between the two groups was also statistically different (*t* = 2.736, *P* = 0.007) (Figure 2B).

## Discussion

Clinical guidelines suggest that drug treatment is the preferred treatment for school-aged children with ADHD (10), and the side effects of most drugs are mild and gradually tolerated. However, parents, especially the parents of the younger age group, are still worried about the potential side effects, causing a low acceptance of and adherence to pharmacological intervention (47). Further, given that some parents might not be able to take part in field parent training due to varieties of reasons while children participated in GEF training, we launched the OPT course. We hypothesize that a program combing the traditional field intervention and online interventions could improve the effects of intervention. To our knowledge, this study demonstrates for the first time that traditional field intervention in conjunction with digital health technology has been successfully applied in both the screening and treatment of ADHD. In this RCT, all participants of online training courses are children's parents. We investigated the children's core ADHD symptoms, EF, behavioral function, and parental pressure through parent report questionnaire data and neurophysiological experiment (Go/No-Go task) at pre-treatment (T1) and post-treatment (T2, after 8 weeks). The benefits of GEF–OPT intervention can be clearly observed in the parents' and teachers' reported reduction of children's core ADHD symptoms and learning problem as well as improvements in EF with a lower level of parental distress in the intervention group at T2. This investigation indicates that the GEF-OPT training program could be a convinced choice of non-pharmacological intervention for younger school-aged ADHD children.

Our GEF-OPT programs combine two training programs that cover a range of symptoms in ADHD. Inattentive symptoms in individuals with ADHD occur due to the lack of sustained effort over time, whereas hyperactivity and impulsiveness originate from the delay aversion and the lack of future sight that is a consequence of altered time perception (48). The differences in long and short time duration perception could be followed with neural correlation. Beyond its core symptoms, ADHD comprises a range of higher-level executive dysfunctions, including deficits in response inhibition, planning, working memory, interference control, and error correction (49). As a consequence, many children with ADHD have trouble in forgetting and impairment in planning. Studies have shown that increased engagement in cognitively challenging activities could promote brain development as well as improve core symptoms of ADHD (50). Considering the participation and interest, our GEF training used functional tasks to target multiple EF components to promote neural and cognitive growth.

Significant functional improvements brought by GEF-OPT are shown in BRIEF scores, including inhibition and emotional control, the metacognition index consisting of working memory, planning/organization, monitor, and total score. The possible reason may be that after a children's training course, we would start the corresponding OPT courses. Parents knew the content of EF courses, conducted practice, and followed behavioral management training at home. For example, children were asked to do tasks in a timetable including EF trainings such as visual tracking task, cancellation test, and other tasks, homework, and real-life activities (e.g., daily chores) under the guidance of parents. Parents used behavior management strategies such as obey training, positive reinforcement method, and token economy to enable children to make positive responses and choices. After that, parents could recognize that the children's behavior was getting better during these processes.

On the other hand, results showed insignificant improvements in the task shifting, initiation, and organization of materials, which were parts of behavioral flexibility and planning and reflect an individual's ability to carry out a certain task independently (51). This may be for the following reasons: our GEF training is a form of group interaction, designed to strengthen the child's ability to hold and manipulate multiple pieces of information, to process information flexibly and the child's team skills. The program was carried out in strict accordance with the study protocol by qualified professionals. The severity of symptoms across the involved children was not identical, and we did not require parents to keep daily completion records in parent–child family tasks. This is why the effect of similar at-home parental training was not as notable. In addition, the duration of training time in each EF lesson might not be enough. Qian et al. (20) reported that the second round of EF training in ADHD students was well-accepted and had positive effects in a 1-year follow-up; this is because children's EF was enhanced by structured, repeated training that extended to early adulthood or even older. Thus, it would be necessary to do fidelity checks and increase the time duration of GEF training as needed.

Gioia et al. (52) proposed that it should be a combination of neurophysiological experiments and ecological assessment tools that fully reflects the subject's EF level. Therefore, the Go/No-Go task was employed to investigate response inhibition, which is a fundamental aspect of every organized cognitive or behavioral response. We were able to find an improvement in the RC and RT of both groups. This was similar to the findings by Monden et al. (53) who found that performance was significantly improved in the post-drug treatment session. Although our intervention was not pharmacological, it showed effectiveness. Defective inhibition processes profoundly affect daily life, leading to impulsive behavior, which is usually detrimental for an individual (54) and has been strongly associated with ADHD. Our research provides a reference for improving inhibition to suppress impulsivity.

We found changes in only a few subscales in the WFIRS-P. One possible explanation of this outcome was that our broad intervention program might have trained all these functions to some extent, leading to a significant improvement of part or overall functions—as found on the learning and school function and total score of WFIRS-P—but not enough for apparent changes on separate functional subscales (55).

As predicted, we found that there were significant differences in parenting distress, dysfunctional interaction, the difficult child, and the total score of PSI between intervention and waitlist groups. This result extended the findings of Franke et al. (33) by offering both EF intervention and using online technology to carry out parent training in families of younger students; in contrast, the former study afforded online parenting intervention only. We demonstrate that the GEF-OPT program frees parents from traffic and time constraints. As a result, this program not only increased the involvement of parents but also increases the efficiency of training lessons. As expected, the parental involvement in this study is higher compared to the traditional GEF program, and the attendance rates for each session were close to 100%. In addition, pediatricians could give precise guidance to families directly. Taken together, this study demonstrates that GEF-OPT offered by professional pediatricians can support parents in managing the stress of raising a school-aged child with ADHD and enhance parent–child communication.

## Strengths and Limitations

The GEF-OPT program provided a multimodal treatment of GEF-OPT for children and parents. This treatment addressed important areas of functional impairment in school-aged students and was led by healthcare professionals. The program reduced barriers for taking part in the intervention and facilitated collaborative treatment efforts with good short-term effects.

The limitation in this study should be noted. This is a short-term effect study without long-term follow-up, so we cannot know whether the intervention can produce long-term improvements. Further study will extend the follow-up time. Additionally, the results would be more robust if the control group took part in a more traditional face-to-face parent–child training intervention. We are planning to improve our study design and gather more evidence to confirm the benefits of the GEF-OPT program to ADHD children in the future.

## Conclusion

In summary, our study provides an evidence of the effectiveness of the GEF-OPT program in decreasing school-aged students' core ADHD symptoms, mitigating executive deficits, and improving learning ability and parental well-being. These findings highlight the potential benefits of the combination of field and online trainings in ADHD intervention.

## Data Availability Statement

The original contributions presented in the study are included in the article/Supplementary Material, further inquiries can be directed to the corresponding authors.

## Ethics Statement

The studies involving human participants were reviewed and approved by Ethics Committee of Shanghai Children's Hospital. Written informed consent to participate in this study was provided by the participants' legal guardian/next of kin. Written informed consent was obtained from the individual(s), and minor(s)' legal guardian/next of kin, for the publication of any potentially identifiable images or data included in this article.

## Author Contributions

LC, GY, and YW contributed to the conception and design of the study. LC, PZ, and LP designed the intervention plan and participated in the training program. CM, LP, and LS contributed to data collection and organization. LC, PZ, and DW performed the statistical analyses and wrote the first draft of the manuscript. All authors contributed to manuscript revision, read, and approved the submitted version.

## Funding

This work was supported by the project of the Shanghai Health and Hygiene Commission on Aging and Maternal and Child Health (2020YJZX0203), a 3-year action plan for the construction of Shanghai public health system (GWV-10.1-XK19,GWV-10.1-XK14), Shanghai Shenkang Hospital Development Center Critical Disease Multi-center Clinical Research Project (SHDC2020CR1047B), Medical Guidance Science and Technology Support Project of Shanghai Science and Technology Commission (19411969000), and Shanghai Natural Science Foundation Project (19ZR1477700).

## Conflict of Interest

The authors declare that the research was conducted in the absence of any commercial or financial relationships that could be construed as a potential conflict of interest. The handling editor FL declared a shared parent affiliation with the authors at the time of the review.

## Publisher's Note

All claims expressed in this article are solely those of the authors and do not necessarily represent those of their affiliated organizations, or those of the publisher, the editors and the reviewers. Any product that may be evaluated in this article, or claim that may be made by its manufacturer, is not guaranteed or endorsed by the publisher.
