# Supplementary material for: Effects of Combing Group Executive Functioning and Online Parent Training on School-Aged Children With ADHD: A Randomized Controlled Trial
Source: Front Pediatr. 2022 Feb 11;9:813305. doi: 10.3389/fped.2021.813305 (PMC8874140; doi:10.3389/fped.2021.813305)
Supplement: Supplementary file 1 [file Data_Sheet_1.pdf]

Supplement 1. Contents of GEF-OPT

Week	Targeted executive function	Part of group executive function training	Online parent training
1	Sustained attention	Commitment: Each child was asked to tell a class rule and then wrote it down or express with pictures in the notebook.<br><br>Visual tracking: The therapist took out three playing cards and put them face up in a row, and asked children to choose one (for example, spades A). Then the therapist put them back to its original position, asked children to focus on the card, and moved the card quickly from side to side. After several moves, children were asked to point out the position of spades A from the three playing cards. The number and type of cards would be changed.	Knowledge about ADHD and methods of family attention training
2	Planning and time management	Schedule: The therapist taught children planning and time management skills and gave each child a timetable as well as asked them to formulate the time they spend on necessary events and other activities for the following week (homework, tutoring class, extracurricular activities, etc.). Children were required to complete the weekly schedule.	Help children manage time and supervise them to complete each task according to the schedule
3	Organization skills	Room and desk organization: Children should be first asked to distinguish clean and cluttered room and desk. The therapist used teaching aids to classify and organize possessions in the room and study with children. Homework was to tidy up the room and desk, and complete a task list for hosting a birthday party.	Learn to mobilize children's enthusiasm and praise them in time
4	Inhibition	Simon says: One child acted as Simon and gave instructions to other children (nodding, stomping, touching nose, etc.). When he started with “Simon says”, the rest of children needed to follow instructions, otherwise they should keep still.	Learn behavioral strategies such as positive reinforcement and punishment to manage conduct problems

5	Working memory	Sherlock: The therapist gave out 8 cards with arrows of different clues (daily necessities, fruits, animals, clothing, etc.). Children needed to remember the evidence on the cards. Then the therapist turned the card face down and picked up the doll. The doll moved according to the arrow and the number of steps on the card. If the child answered correctly and the card the doll stayed on was turned over, the child would get this card.	Strategies for effective learning skills and communication with teachers
6	Spatial intelligence	Matchmaker: The therapist gave a card surrounded by 10 blocks (from easy to difficult). Children needed to flip 5 long blocks in the shortest time to match the corresponding pattern.	Guidelines for giving effective instructions
7	Cognitive flexibility	My first journey: The therapist taught children to understand the map of China. Four city tickets were randomly selected on the table. Each child had another four city tickets, then took turns rolling the dice, and chose the route according to the color of the dice and city tickets. When the arrival city was the same as the four tickets on the table, the child could get the ticket of the stated characteristics of the city.	Games of improving parent-child relationship and methods for stress management
8	Consolidate and summarize	Consolidate and reinforce the poorly-performed projects completed before. Children shared their positive changes and received rewards.	Questions and answers<br><br>Review and identified obstacles resolution

Supplement 2. Demographic characteristics of the intervention group and the waitlist group

Variable	Intervention (n=73)	Waitlist (n=72)	t/χ^2	*P*
**Age (years), mean±SD**	7.10±0.47	7.04±0.61	0.666	0.506
**IQ, mean±SD**	97.01±17.31	96.36±12.23	0.262	0.794
**Gender, n (%)**			0.667	0.414
Boy	57 (78.1)	52 (72.2)		
Girl	16 (21.9)	20 (27.8)		
**ADHD subtype, n (%)**			1.002	0.606
Inattentive	45 (61.6)	42 (58.3)		
HI	8 (11.0)	12 (16.7)		
Combined	20 (27.4)	18 (25.0)		
**Comorbidity, n (%)**				
ODD	15 (20.5)	13 (18.1)	0.145	0.704
Anxiety and depression	2 (2.7)	4 (5.6)	0.725	0.395
**Family structure, n (%)**			1.242	0.265
core family	40 (54.8)	46 (63.9)		
non-core family	33 (45.2)	26 (36.1)		
**Family annual income, yuan n (%)**			2.687	0.261
~100,000	9 (12.3)	10 (13.9)		
100,000-200,000	19 (26.0)	27 (37.5)		
200,000~	45 (61.6)	35 (48.6)		

**Parental relationship, n (%)**			0.090	0.764
Harmony	49 (67.1)	50 (69.4)		
General	24 (32.9)	22 (30.6)		
**Father's education, n (%)**			1.602	0.449
College~	12 (16.4)	16 (22.2)		
High school-College	48 (65.8)	40 (55.6)		
~Junior high school	13 (17.8)	16 (22.2)		
**Mother's education, n (%)**			0.510	0.775
College~	9 (12.3)	9 (12.5)		
High school-College	53 (72.6)	49 (68.1)		
~Junior high school	11 (15.1)	14 (19.4)		
**Parent-child communication time, n (%)**			0.222	0.638
<3d/w	2 (2.7)	3 (4.2)		
≥3d/w	71 (97.3)	69 (95.8)		
**Parent-child outdoor activities, n (%)**			2.846	0.092
<3d/w	43 (58.9)	52 (72.2)		
≥3d/w	30 (41.1)	20 (27.8)		
**Children’s exposure to electronic screens time, n (%)**			5.239	0.073
1h/d~	38 (52.1)	27 (37.5)		
0.5h/d-1h/d	19 (26.0)	17 (23.6)		
~0.5h/d	16 (21.9)	28 (38.9)		

NOTE. ADHD: Attention deficit hyperactivity disorder; IQ: Intelligence quotient; HI: Hyperactive-impulsivity; ODD: Oppositional-defiant disorder; SD: Standard deviation.

Supplement 3.Effects of GEF-OPT by SNAP-IV scales.

Scales	Intervention group (n=73)		Waitlist group (n=72)		*F*	*P*	*d* [95% CI]
	Pre	Post	Pre	Post			
**SNAP-IV, Parent Rated**							
Inattentive	15.66±3.99	14.70±4.35	15.86±4.03	16.03±2.93	5.17	0.024	0.27 [-0.06,0.60]
HI	11.47±5.19	9.85±5.30	12.58±5.52	10.69±5.10	0.01	0.913	-0.41 [-0.69,-0.14]
ODD	8.53±4.78	7.03±4.39	8.93±3.94	8.53±4.41	4.55	0.035	0.27 [-0.03,0.57]
Total score	35.66±9.79	31.58±11.32	37.38±10.74	35.25±10.44	3.34	0.070	0.06 [-0.21,0.33]
**SNAP-IV, Teacher Rated**							
Inattentive	16.19±2.99	14.56±3.96	15.90±4.05	16.06±2.74	13.23	<0.001	0.53 [0.24,0.82]
HI	12.74±4.10	10.64±4.79	12.46±4.53	11.28±4.16	2.59	0.110	-0.09 [-0.36,0.18]
ODD	9.60±3.89	7.86±3.93	8.92±3.79	8.90±3.62	13.05	<0.001	0.53 [0.28,0.78]
Total score	38.53±7.76	33.07±10.06	37.28±10.54	36.24±9.48	14.76	<0.001	0.43 [0.17,0.69]

NOTE. All data are shown as mean ± SD. SNAP-IV: Swanson Nolan and Pelham, Version IV Rating Scale; HI: Hyperactive-impulsivity; ODD: oppositional-defiant disorder; SD: Standard deviation.

Supplement 4. Effects of GEF-OPT by BRIEF scales

Scales	Intervention group (n=73)		Waitlist group (n=72)		*F*	*P*	*d* [95% CI]
	Pre	Post	Pre	Post			
**BRIEF**							
Inhibition	19.88±5.44	17.21±4.37	19.85±4.12	19.44±4.58	21.85	<0.001	0.69 [0.43,0.95]
Shift	13.00±2.76	13.07±2.69	13.85±2.53	13.60±2.70	0.00	0.982	0.00 [-0.33,0.33]
Emotional Control	17.36±4.80	15.82±4.27	18.36±4.47	17.78±4.59	7.24	0.008	0.33 [0.11,0.55]
Initiate	15.33±2.98	14.79±2.80	15.29±2.84	14.99±3.16	0.34	0.562	0.06 [-0.21,0.33]
Working Memory	22.47±3.60	21.22±4.12	23.54±3.46	23.32±3.80	6.81	0.010	0.27 [0.01,0.54]
Planning/Organization	25.71±4.67	24.42±4.62	25.63±4.84	25.50±4.50	5.10	0.025	0.32 [0.09,0.56]
Organization of Materials	12.08±2.31	11.52±2.51	12.00±2.44	11.93±2.17	2.89	0.091	0.28 [0.03,0.53]
Monitor	19.53±3.14	17.92±3.09	19.64±2.95	19.01±2.86	7.45	0.007	0.34 [0.05,0.63]
BRI	50.23±10.41	46.10±8.68	52.06±9.17	50.82±10.41	14.77	<0.001	0.42 [0.21,0.63]
MI	95.12±13.38	89.88±14.15	96.10±15.03	94.75±14.93	7.39	0.007	0.30 [0.07,0.53]
Total score	145.36±20.71	135.97±19.83	148.15±23.23	145.57±24.33	12.67	0.001	0.32 [0.09,0.54]

NOTE. All data are shown as mean ± SD. BRIEF: Behavior Rating Inventory of Executive Function-Parent Form; BRI: Behavioral Regulation Index; MI: Metacognition Index; SD: Standard deviation.

Supplement 5. Effects of GEF-OPT by WFIRS-P and PSI scores

Scales	Intervention group (n=73)		Waitlist group (n=72)		*F*	*P*	*d* [95% CI]
	Pre	Post	Pre	Post			
**WFIRS-P**							
Family	8.03±4.14	6.84±3.61	8.50±3.64	7.69±3.81	1.43	0.233	0.11 [-0.25,0.48]
Learning and School	6.25±3.50	5.23±2.94	5.26±3.12	6.14±3.39	8.52	0.004	0.60 [0.27,0.94]
Life Skills	9.55±3.86	9.18±3.32	9.40±4.03	9.64±4.85	0.82	0.365	0.17 [-0.15,0.48]
Self-Concept	2.26±1.91	2.07±1.78	2.10±1.46	2.07±1.35	0.03	0.855	0.11 [-0.31,0.53]
Social Activities	5.81±3.69	4.85±2.99	5.94±2.87	5.63±3.42	2.05	0.155	0.20 [-0.21,0.61]
Risky Activities	2.91±2.09	2.64±2.25	3.18±1.82	2.97±2.05	0.35	0.553	0.05 [-0.30,0.39]
Total score	34.80±12.79	30.81±11.47	34.39±12.50	34.14±10.49	6.99	0.009	0.30 [0.03,0.56]
**PSI**							
Parenting distress	27.78±4.87	25.16±4.17	28.79±4.38	28.51±4.03	28.45	<0.001	0.73 [0.43,1.03]
Dysfunctional interaction	28.78±5.98	24.99±4.77	28.72±5.93	28.29±4.41	37.72	<0.001	0.98 [0.67,1.29]
Difficult child	27.74±6.14	25.52±4.96	27.21±5.54	27.08±5.38	14.39	<0.001	0.91 [0.65,1.16]
Total score	84.30±13.11	75.67±10.23	84.72±13.71	83.89±11.27	48.75	<0.001	1.20 [0.89,1.50]

NOTE. All data are shown as mean ± SD. WFIRS-P: WEISS Functional Impairment Scale-Parent form; PSI: Parent Stress Index; SD: Standard deviation.
